# A pyrimidine metabolism-related signature for prognostic and immunotherapeutic response prediction in hepatocellular carcinoma by integrating analyses

**DOI:** 10.18632/aging.205663

**Published:** 2024-03-15

**Authors:** Shihang Zhang, Ouyang Qin, Shu Wu, Huanming Xu, Wei Huang, Song Hailiang

**Affiliations:** 1 Department of General Surgery, Dalang Hospital, Dongguan, Guangdong, P.R. China; 2 Affiliated Dongguan Hospital Southern Medical University (Dongguan People’s Hospital) Dongguan Guangdong, Guangdong, P.R. China; 3 Department of Hepatic-Biliary-Pancreatic Surgery, The Affiliated Hospital of Guizhou Medical University, Guiyang, P.R. China

**Keywords:** hepatocellular carcinoma, pyrimidine metabolism, prognosis, immunotherapy, biomarker

## Abstract

Background: Hepatocellular carcinoma (HCC), with discouraging morbidity and mortality, ranks as one of the most prevalent tumors worldwide. Pyrimidine metabolism is a critical process that regulates DNA and RNA synthesis in cells. It is imperative to investigate the significance of pyrimidine metabolism in liver cancer.

Methods: Transcriptome and clinical data were downloaded from the TCGA database and the GEO database. The genes related to pyrimidine metabolism were sourced from the MSigDB. The pyrimidine metabolism-related signature (PMRS) was constructed through Cox regression and Lasso regression and then verified in the external validation set from the ICGC database. Functional enrichment, immune infiltration analysis, drug sensitivity, and Immunophenoscore (IPS) were further implemented to predict the response to immunotherapy. The role of PMRS in the malignant phenotype of hepatocellular carcinoma was explored by conducting a series of *in vitro* experiments.

Results: Our study developed a four-genes PMRS which demonstrates a substantial correlation with the prognosis of HCC patients, serving as an independent predictor in clinical practice. The result of risk-stratified analysis yielded evidence that low-risk patients experienced more favorable clinical outcomes. The nomogram exhibited remarkable prognostic predictive value. The subsequent results revealed that low-risk patients manifested a more promising response to immunotherapy. Moreover, the results of cell experiments demonstrated that the downregulation of DCK markedly inhibited the malignant phenotype of hepatocellular carcinoma.

Conclusions: Our pyrimidine metabolism-centered prognostic signature accurately predicts overall survival, immune status, and treatment response in hepatocellular carcinoma (HCC) patients, offering innovative insights for precise diagnosis, personalized treatment, and improved prognosis.

## INTRODUCTION

Liver cancer, characterized by discouraging morbidity and mortality, is one of the most frequent lethal gastrointestinal malignancies worldwide [1, 2]. Epidemiologically speaking, hepatocellular carcinoma constitutes roughly 90% of all incidences of primary liver cancer [3, 4]. Owing to the asymptomatic nature of early-stage such as abdominal pain, jaundice, weight loss, fatigue, and fever, patients diagnosed with HCC are regularly diagnosed at an advanced stage, compromising their eligibility for curative resection and resulting in suboptimal outcomes [5]. Risk factors for developing HCC include chronic hepatitis B or C infection, excessive alcohol consumption, non-alcoholic fatty liver disease, exposure to aflatoxins, and obesity [6]. The immune microenvironment exerts a vital influence on the initiation and progression of HCC, while distinct immune features dependent on various etiologies have been delineated [7, 8]. Recently, the usage of immunotherapy has revolutionized the management of HCC [9]. Targeting the programmed cell death and programmed cell death ligand-1 (PD-1/PD-L1) axis has achieved unprecedented success in HCC, but it also faces great challenges, with its low remission rate still to be solved [10]. It is crucial to identify biomarkers that can accurately predict response to immunotherapy for obtaining improved prognoses in patients with HCC.

Tumor metabolic reprogramming refers to the changes in metabolism that occur in tumor cells to facilitate their uncontrolled growth and proliferation [11, 12]. This process involves a variety of alterations in metabolic pathways such as glycolysis, glutaminolysis, lipid metabolism, oxidative phosphorylation, and pyrimidine metabolism.

Pyrimidines are essential components of nucleic acids and play pivotal roles in DNA synthesis and cell proliferation. Pyrimidine metabolism is a complex process that involves de novo synthesis, salvage pathways, degradation, and interconversion of pyrimidine nucleotides [13, 14]. Aberrations in pyrimidine metabolism have been implicated in several diseases, including cancer. Recent studies have shown that dysregulation of pyrimidine metabolism is involved in the pathogenesis of malignancy. Alterations in key enzymes and transporters involved in pyrimidine metabolism have been found to be involved in tumor progression and resistance to chemotherapy [15–17]. For instance, it has been reported that the oncogenic protein ubiquitin-conjugating enzyme E2T (UBE2T) is elevated and may enhance pyrimidine metabolism by encouraging Akt K63-linked ubiquitination, thereby facilitating the progression of HCC [18]. Blocking the pyrimidine synthetic rate-limiting step enzyme CAD or the essential downstream enzyme DHODH suppressed the ability to survive and regenerate glioblastoma stem cells (GSCs) [19]. Given these findings, targeting pyrimidine metabolism could be an attractive approach for cancer treatment including HCC.

Therefore, based on the online databases, we explored and established a PMRS for this investigation. Integrated analyses were subsequently conducted to evaluate the predictive ability of this signature for HCC patients’ survival, sensitivity to chemotherapy, and response to immunotherapy, in addition, the PMRS was validated through cell experiments. In general, our research may yield fresh perspectives into the diagnosis and medical management of HCC.

## MATERIALS AND METHODS

### Data acquisition

We retrieved the data of expression and clinical information of 374 HCC cases, along with 50 normal tissue samples from the TCGA data portal (https://portal.gdc.cancer.gov/projects/TCGA). The GEO database (http://www.ncbi.nlm.nih.gov/geo/) provided the microarray dataset GSE14520 (N=425). To evaluate the prognostic performance of PMRS, an independent validation cohort was utilized, consisting of 223 tumorous samples and 202 normal tissue samples obtained from the ICGC database (https://dcc.icgc.org/releases/current). For the prediction of immunotherapeutic response, we downloaded and included the IMvigor210 cohort in our study. Gene sets related to pyrimidine metabolism were searched for in the Molecular Signatures Database (MSigDB) (https://www.gsea-msigdb.org/gsea/index.jsp), which were subsequently utilized in the investigation. The flow chart displayed the flow of this study.

### Identification and enrichment analysis of the differentially expressed PMRGs

Differentially expressed pyrimidine metabolism-related genes between tumor and normal tissues were screened out with the criteria through the ‘limma’ R package [20]. The ‘clusterProfiler’ R package was utilized for Gene Ontology (GO) and Kyoto Encyclopedia of Genes and Genomes (KEGG) enrichment analyses of those differentially expressed genes. (https://www.liebertpub.com/doi/10.1089/omi.2011.0118).

### Consensus clustering of the PMRGs

Based on the differentially expressed PMRGs, the R package ‘ConsensusClusterPlus’ was utilized to execute consensus clustering and determine discrete clusters related to pyrimidine metabolism [21]. To ensure the robustness and stability of our results, we replicated this process 1000 times. Principal component analysis (PCA) was employed to classify PMRGs into clusters. According to the pertinent R package, the OS possibility of PMRG clusters was compared.

### Construction and validation of the PMRS

We sequentially completed the Cox and Lasso regression to seek out the optimal independent predictive signature. Furthermore, we computed each sample’s risk score using the subsequent formula: Risk Score = (Coef_1_ * Exp_1_ +……+ Coef_n_* Exp_n_). Patients were separated into two categories based on the median value of the anticipated risk scores, which served as the threshold. The Kaplan-Meier analysis was employed to compare the probability of survival within these two categories. Time-dependent ROC analysis was performed through the R package “timeROC”. Survival curves of entire GEO and ICGC cohorts were used to examine the capacity of PMRS to distinguish between groups with different risks in terms of prognosis. Furthermore, stratified analysis was performed to evaluate the prognostic significance of PMRS within segments distinguished through clinical features.

### Establishment and assessment of the pyrimidine metabolism-related nomogram

The independent prognostic factors were determined by the accomplishment of Cox regression upon PMRS along with its related clinical parameters. The nomogram was subsequently devised to calculate the survival probabilities of HCC patients. Utilizing calibration and time-dependent ROC, we assessed the reliability and quality of the nomogram. Additionally, employing DCA, we contrasted the cumulative effect of the entire nomogram with that of this model incorporating solely clinical measures.

### Functional enrichment analysis

With the annotated gene set obtained from MSigDB, the gene set enrichment analysis (GSEA) was carried out to identify the differences in linked pathways across various risk categories [22].

### Evaluation of immunotherapy efficacy and investigation of the immune profile

The ESTIMATE algorithm is a computational method used to estimate the presence and proportion of immune and stromal cells within tumor tissue samples [23]. Based on the theory of linear support vector regression, the method known as CIBERSORT evaluates 22 immune cell type fractions from mass tumor sample data regarding expression using an array of reference gene expression matrices [24, 25]. The comparison of the immune checkpoint, human leukocyte antigen (HLA), and immune function between distinct risk groups was consecutively conducted. The IPS is determined by four main gene categories that decide the degree of immunogenicity, and it is acquired through unbiased analysis utilizing machine learning techniques. In this work, we used IPS data to assess the prospective immunotherapy response. The Submap method was also used to forecast how various risk groups would respond to anti-PD-1 and anti-CTLA-4 treatment [23]. Furthermore, we also utilized clinical and immunotherapy data derived from the ‘IMvigor210CoreBiologies’ to assess the PMRS’s susceptibility to anti-PD-1 treatment in our analysis.

### Identification of drug sensitivity

We downloaded the “DrugPredict.tsv” file and estimated the sensitivity score for common drugs and small chemical molecules in the HCC therapy. Additionally, we mined the correlation between PMRS genes and drug sensitivity using the CellMiner database [26] (https://discover.nci.nih.gov/cellminer/home.do).

### Validation of the PMRS gene by single-cell RNA analysis

The Tumor Immune Single-cell Hub (TISCH) database (http://tisch.comp-genomics.org/home/) is an open-access platform that focuses on the single-cell sequencing data of the tumor microenvironment [27]. It provides comprehensive and detailed information to help researchers better understand the immune responses in tumors and corresponding single-cell transcriptome data [28]. The establishment of this database provides an important resource for further exploring tumor immunotherapy. We exploited this collection of data to figure out the association between the levels of expression of four genes responsible for establishing the PMRS and the infiltration of diverse immune cell types.

### Cell culture and transfection

Hepatocellular carcinoma cell lines MHCC-97H and HepG2 were obtained from the American Type Culture Collection (USA). These cells were cultured at 37° C, 5% CO2 with 10% FBS and 1% penicillin/streptomycin in RPMI-1640. Lipofectamine 3000 was applied to transfect cells with siRNAs. The three siRNA sequences for DCK are as follows: (siRNA-1) 5’- GCCUGUCUCAGUCGAAUAATT – 3’, (siRNA-2) 5’- GCCUUGAAUUGGAUGGAAUTT – 3’ and (siRNA-3) 5’- GCUCAAAGAUGCAGAGAAATT – 3’.

### Transfection efficiency

The efficiency of siRNA transfection was evaluated by Western blot assay. Cellular total proteins were isolated with RIPA lysate buffer and quantified with the bicinchoninic acid assay. Thirty micrograms of protein were loaded onto a polyacrylamide gel and separated by electrophoresis, then transferred to PVDF membranes. Following blocking with 5% BSA, the membranes were incubated overnight at 4° C with specific primary antibodies against DCK (1:1000, Proteintech, China) and GAPDH (1:1000, Proteintech). On the following day, the membranes were washed with TBST three times (10 min per time) and then incubated with a secondary antibody (1:3000) for 2 hours at room temperature. Signal intensities were quantified by the fully automatic chemiluminescence image analysis system.

### CCK-8 assay

When their siRNA transfection level exceeded 90%, MHCC-97H and HepG2 cells were digested followed by inoculation onto 96-well culture plates with 2103 per well, and 3 duplicate wells were created for each group. The cells were then cultivated in a 5% CO2 incubator at 37° C and examined using the CCK-8 kit at various intervals of 0h, 24h, 48h, 72h, and 96h. (Beyotime Biotechnology, Shanghai, China).

### Transwell assay

The lower chamber was covered with 700 μL complete medium. Then, 5×10^4^ cells of each group in 200 μL serum-free medium were seeded in the upper chamber, and then cultured in an incubator (37° C, 5% CO2) for 24h. Next, the cells underwent fixation with 4% paraformaldehyde for a period of 20 minutes, after which they were subjected to staining with crystal violet for 30 minutes. Use sterile cotton to wipe off the cells remaining in the upper chamber slightly. The transwells were dried at room temperature and photographed under a microscope.

### Wound healing assay

After the siRNA-transfected cells in the 6-well plate grew to almost 100%, the cells were scratched in the plate with the 200 μL pipetting head and replaced with the serum-free medium to avoid cell growth effects. Subsequently, the image was captured with a microscope at 0h, 24h, and 48h after scratching.

### Statistical analyses

All statistical analyses were performed using R software. Spearman analysis was used to analyze correlation coefficients. For normally distributed continuous data, we used independent sample tests, and for irregularly distributed continuous variables, Mann-Whitney U tests. The data comparison between the three groups was done using one-way ANOVA. For survival analysis, the Kaplan-Meier technique with a two-sided log-rank test was used. Statistical significance was defined as a p-value < 0.05. (*P<0.05, **P<0.01, ***P<0.001, ****P<0.0001).

### Data availability statement

This article features the original research contributions presented by our study. For any additional inquiries, interested parties may contact the corresponding author directly.

## RESULTS

### Flow chart

**Figure 1:**
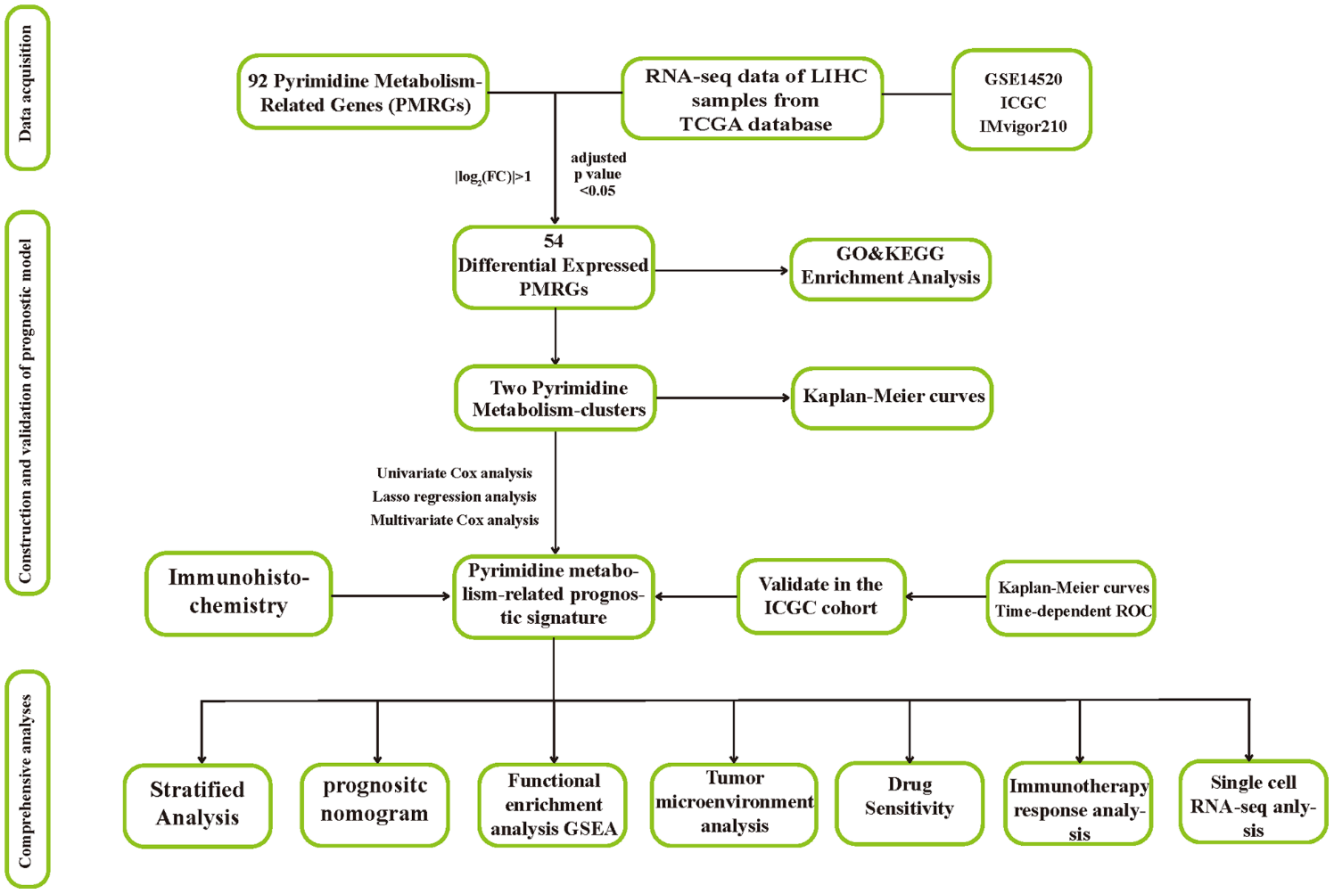
The flow chart of this research.

The flow chart displayed the flow of this study (Figure 1).

### Genetic and transcriptional landscape of PMRGs

**Figure 2:**
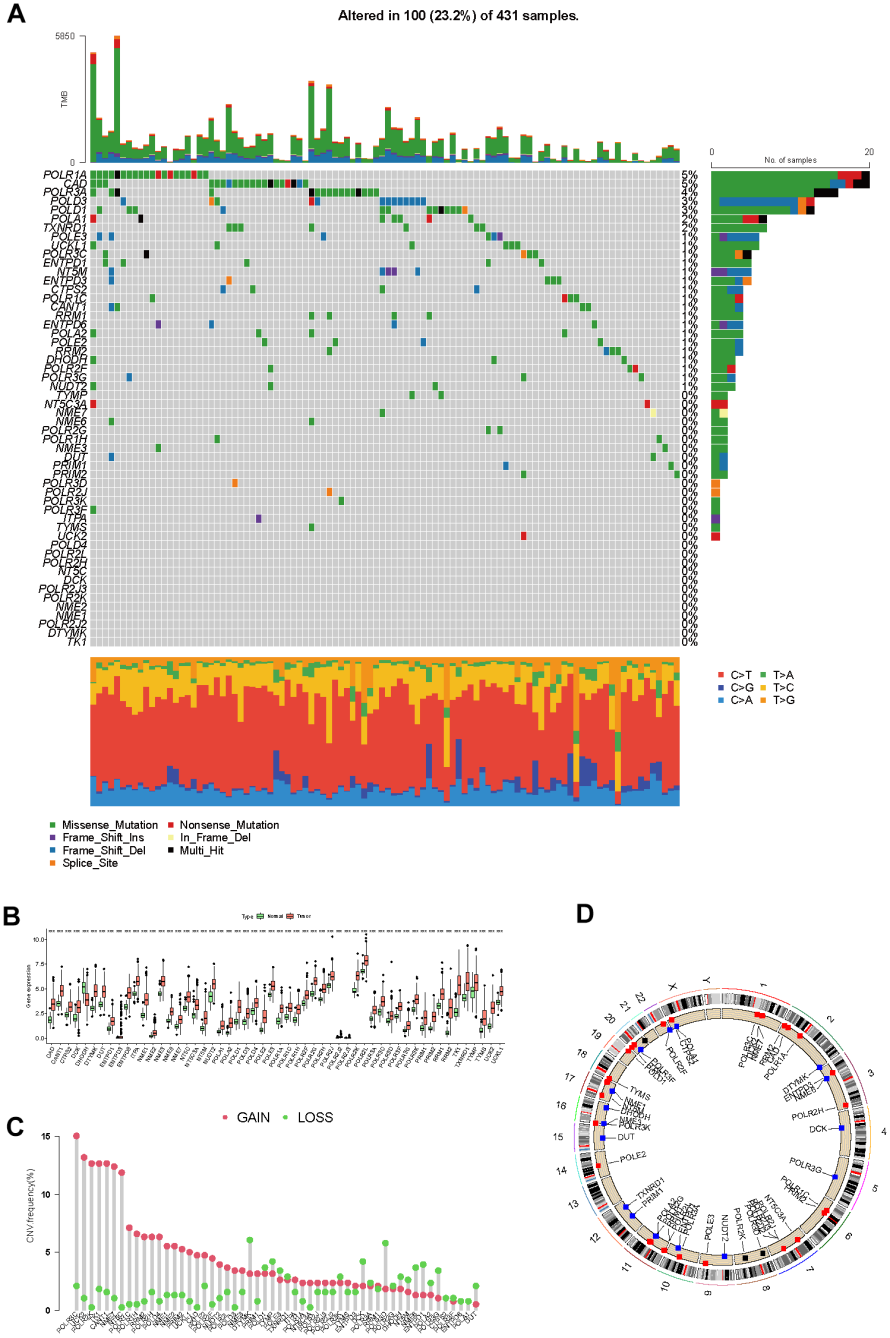
**The genetic and transcriptional landscape of PMRGs.** (**A**) The mutation profile of differentially expressed PMRGs in the TCGA-LIHC cohort. (**B**) The differential expression of PMRGs in HCC between tumor and normal tissues. (**C**) The CNV patterns of 54 PMRGs within HCC. (**D**) The locations of the CNV variations of 54 PMRGs on 23 chromosomes.

From the MSigDB database, we initially collected and cataloged 92 genes associated with pyrimidine metabolism. Subsequently, 54 differentially expressed genes were filtered out by criteria of the absolute value of log_2_(FC) > 1 and an adjusted *P*-value < 0.05. As displayed in the genetic variation analysis, missense mutations were the top common type among the 100 (23.2%) mutation samples out of a total of 431 samples (Figure 2A). RNA polymerase I subunit A (POLR1A), Carbamoyl-phosphate synthetase 2-aspartate transcarbamylase-dihydroorotase (CAD), and RNA polymerase III subunit A (POLR3A) ranked the top three mutant PMRGs. According to the results of differential analysis, there were 53 upregulated genes and 1 downregulated gene (Figure 2B). Additionally, we explored the CNV patterns of 54 PMRGs within HCC. (Figure 2C). Figure 2D depicts the locations of the CNV variations of 54 PMRGs on 23 chromosomes.

### Development of consensus cluster on PMRGs

**Figure 3:**
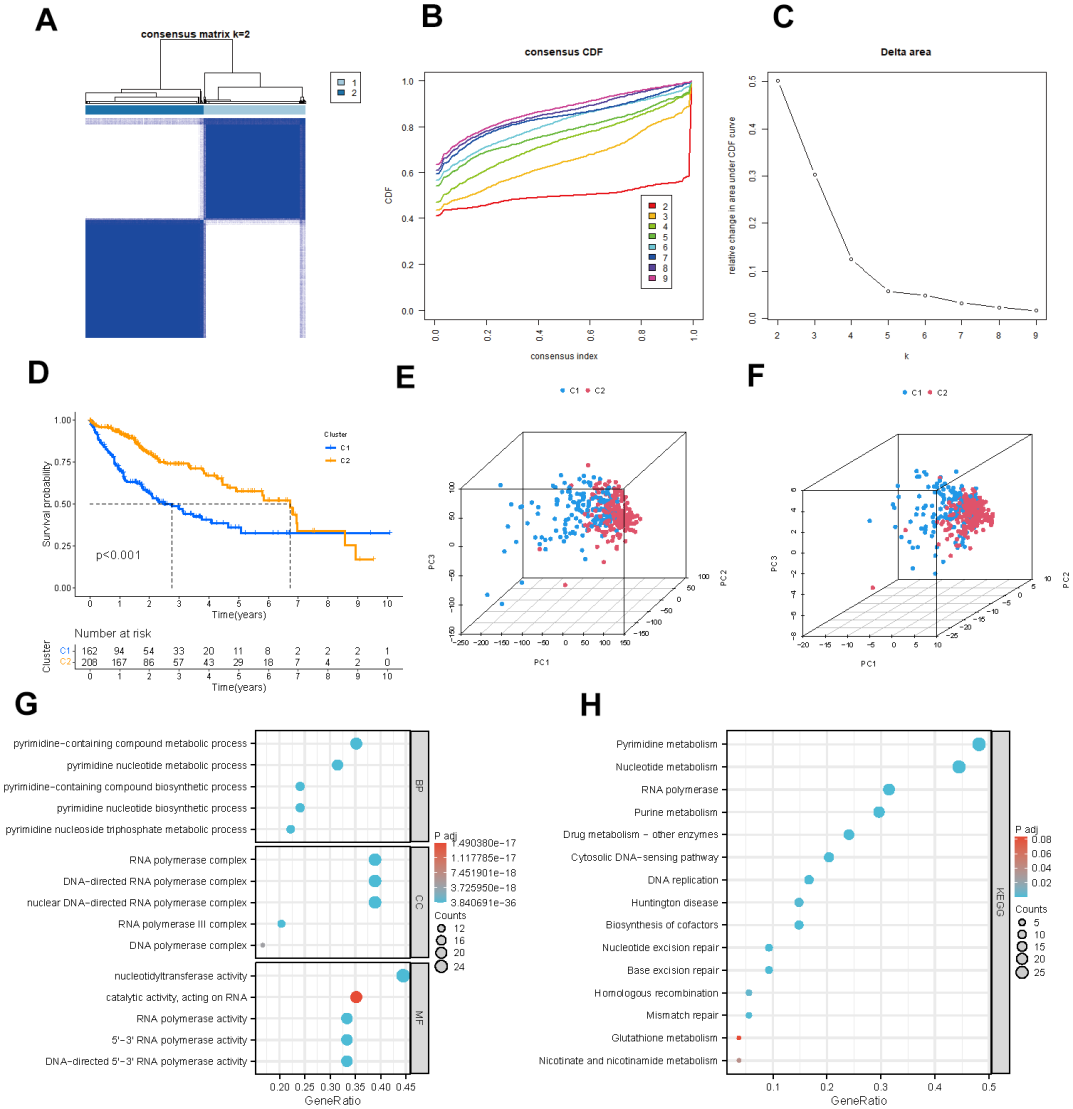
**Identification of potential pyrimidine metabolism-related clusters in HCC patients.** (**A**) The consensus clustering analysis of PMRGs. (**B**) Consensus CDF. (**C**) Delta area. (**D**) Kaplan-Meier survival analysis for different clusters. (**E**, **F**) The PCA plots of the PMRG clusters. (**G**, **H**) GO and KEGG enrichment analysis of differentially expressed PMRGs.

We used a consensus clustering approach to categorize the pyrimidine metabolism-related clusters to examine the expression properties and probable biological features of 54 PMRGs in HCC. According to the analysis of the cumulative distribution function, k=2 demonstrates exceptional clustering elasticity. The intra-cluster correlation is relatively strong while the inter-cluster correlation is low (Figure 3A–3C).

We employed the Kaplan-Meier survival analysis to show a more prolonged overall survival of HCC patients in cluster B (Figure 3D, P <0.001). The PCA graphs revealed a strikingly distinct pattern between clusters for all PMRGs and differentiated PMRGs. (Figure 3E, 3F). Then, functional enrichment analysis on the differentially expressed PMRGs was performed. As the visualization results demonstrated, biological processes (BPs) were predominantly enriched in the pyrimidine−containing compound metabolic process; cellular components (CCs) were significantly enriched in DNA and RNA polymerase complex; molecular functions (MFs) were mainly enriched in nucleotidyltransferase activity and catalytic activity. KEGG enrichment analysis exhibited that these genes were enriched in pyrimidine metabolism and nucleotide metabolism pathway (Figure 3G, 3H).

### Development and verification of the prognostic model related to pyrimidine metabolism

**Figure 4:**
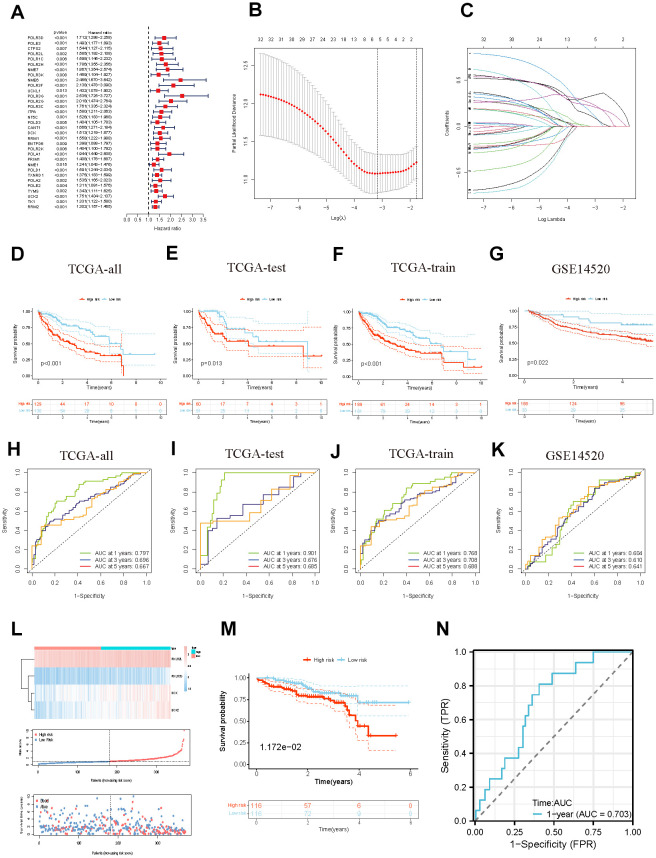
**Identification and validation of the PMRS.** (**A**) Forest plot of the univariate Cox regression. (**B**, **C**) Construction of PMRS. (**D**–**G**) The OS KM curves between different groups respectively in the whole TCGA cohort, test cohort, train cohort, and GSE14520. (**H**–**K**) The time-dependent ROC curves of the PMRS respectively in the whole TCGA cohort, test cohort, train cohort, and GSE14520. (**L**) The risk score and clinical event between the different groups in TCGA cohort. (**M**) The OS KM curves between high- and low-risk groups in the ICGC cohort. (**N**) The time-dependent ROC of the PMRS in the ICGC cohort.

We employed univariate Cox analysis to ascertain genes that exhibit a significant association with the survival of HCC patients (Figure 4A). Moreover, a pyrimidine metabolism-related 4-gene model was constructed to predict prognosis through Lasso and multivariate Cox regression analysis (Figure 4B, 4C). Risk score = (0.5961×POLR2L) + (0.3923×POLR3G) + (0.3057×DCK) +(0.3794×UCK2). On the basis of the cutoff, patients in the LIHC cohort were divided into two categories: high- and low-risk. To evaluate and validate the reliability and accuracy of the model, the TCGA cohort was separated into test and train groups. According to the survival curves, the patients in the high-risk group suffered from poorer prognosis in TCGA cohort and GSE14520 cohort (Figure 4D–4G). The area under curve (AUC) values for the predicted survival rates at 1-, 3-and 5-year were 0.797, 0.696, and 0.667 respectively in TCGA cohort; 0.901, 0.676, and 0.688 in TCGA test group; 0.768, 0.708 and 0.688 in TCGA train group; 0.604, 0.610, and 0.641 in GSE14520 cohort, which demonstrate the extraordinary prognostic predictability of the model (Figure 4H–4K). Figure 4L displays the risk score, clinical factors, and expression of four signature genes between the two risk categories. Furthermore, we developed external validation to evaluate the predictive power of the model utilizing the HCC cohort from the ICGC database. The ICGC cohort was divided into high- (n=116) and low-risk (n=116) groups. The KM survival curve (Figure 4M, P=0.011) revealed that the high-risk group had inferior outcomes than the low-risk group. In addition, the 1-year OS AUC value in the ICGC cohort was 0.703 (Figure 4N).

### Stratified analysis and development of the pyrimidine metabolism-related nomogram

**Figure 5:**
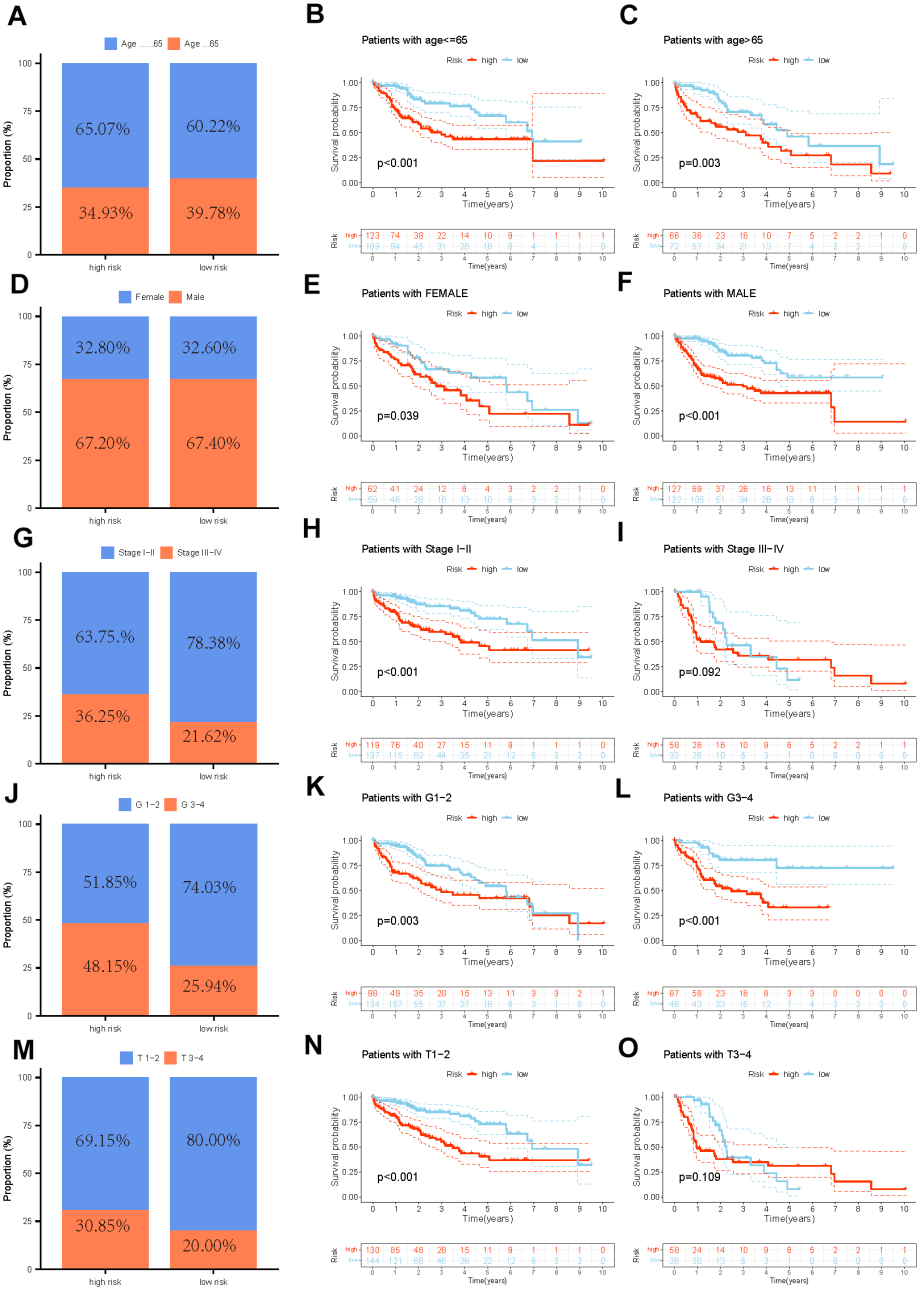
**The distribution features and the OS KM curve of various clinicopathological factors in different risk groups.** (**A**–**C**) Age, (**D**–**F**) Gender, (**G**–**I**) Grade, (**J**–**L**) Stage, (**M**–**O**) T.

**Figure 6:**
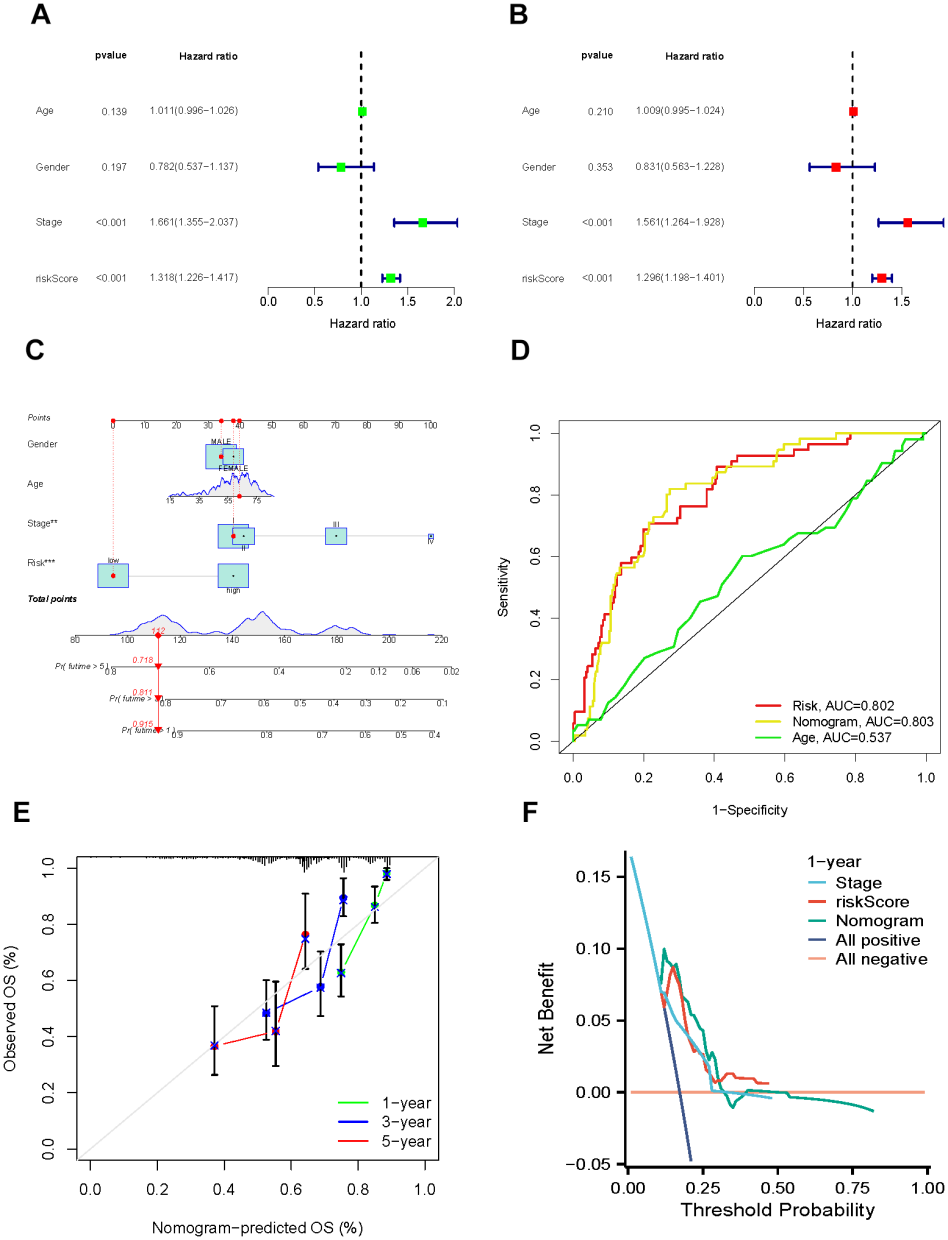
**Establishment and assessment of the nomogram.** (**A**, **B**) Univariate and multivariate regression. (**C**) Nomogram for the prediction of HCC patients’ survival. (**D**) ROC curve of clinical factors and nomogram. (**E**) Calibration curves of the nomogram at 1-, 3-, and 5-years. (**F**) DCA curves of clinical factors and this nomogram.

We segregated individuals into different categories and calculated their overall survival for the stratified analysis to further validate the predictive value of the signature in subgroups with distinct clinical factors. The results of the distribution characteristics of the high- and low-risk categories remained comparable with the overall finding that the low-risk group obtained relatively favorable clinical results. (Figure 5A–5O). Subsequently, both major clinicopathological factors and the gene signature were analyzed employing univariate and multivariate Cox regression models to identify their prognostic significance. As the results displayed, Stage [(HR:1.661, 1.355−2.037, *P* < 0.001) in univariate Cox; (1.561, 1.264−1.928, *P* < 0.001) in multivariate Cox] and risk score [(1.318, 1.226−1.417, *P* < 0.001) in univariate Cox; (1.296, 1.198−1.401, *P* < 0.001) in multivariate Cox] were significantly associated with HCC prognosis and potential to be independent predictors (Figure 6A, 6B). We constructed a comprehensive nomogram with the identified independent prognostic factors to provide quantitative predictions of 1-, 3-, and 5-year overall survival (OS) probabilities in patients with HCC (Figure 6C). The AUC values were 0.802, 0.803, and 0.537 for risk, nomogram, and age respectively, which revealed the accuracy of the predictions (Figure 6D). The calibration curves indicated strong agreement between the actual and predicted overall survival (OS) rates (Figure 6E).

To evaluate the clinical usefulness of the nomogram, we assessed its performance via decision curve analysis (DCA) [29]. Our results manifested that, in comparison to the model relying solely on clinical characteristics, the comprehensive nomogram could lead to increased net benefits and potentially improve the clinical management of the disease (Figure 6F).

### Detection of the immune landscape

**Figure 7:**
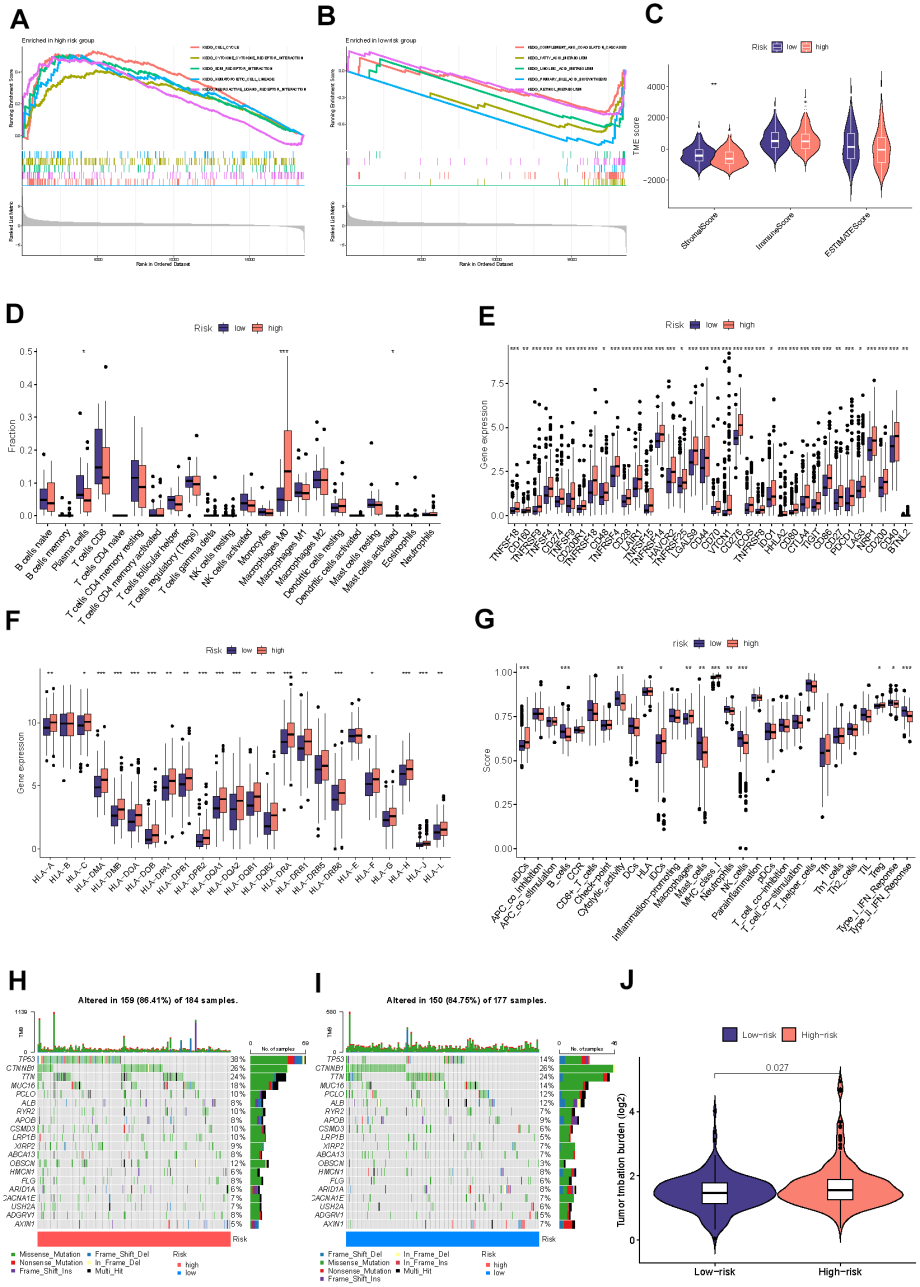
**Detection of the immune profile between different groups.** The GSEA results for the high-risk (**A**) and low-risk (**B**) groups. (**C**) Difference between two risk groups’ stromal, immune, and ESTIMATE scores. (**D**) Comparisons of the fractions of immunocyte infiltration between distinct groups. (**E**) Differential checkpoint gene expression between two groups. (**F**) The differential HLA gene expression between two groups. (**G**) The levels of immune function in the two at-risk categories. The mutational status of the leading 20 genes in the high-risk category (**H**). The low-risk cohort (**I**). (**J**) The TMB concentration in the various categories. (*P<0.05, **P<0.01, ***P<0.001, ****P<0.0001).

To further investigate the biological progress of the two risk groups by conducting GSEA. Our observations suggest that the high-risk group exhibited a significant enrichment of cancer-associated processes, such as cell cycle and ECM receptor interaction. In contrast, the low-risk group demonstrated significant enrichment of complement and coagulation cascades, which are known to function in the immune system (Figure 7A, 7B). The exploration of the immune microenvironment was performed by ESTIMATE and CIBERSORT algorithms. Following the outcomes of the ESTIMATE analysis, the group with the low risk had a significantly higher stromal score. The CIBERSORT analysis revealed that the high-risk group owned a significantly higher proportion of M0 macrophages and activated mast cells, while a higher proportion of plasma cells in the low-risk group (Figure 7C, 7D). These observational findings imply that the tumor growth and prognosis of the different groups may vary in terms of immune cell infiltration. For example, a higher proportion of M0 macrophages may affect antigen presentation and T cell activation, thereby accelerating tumor deterioration; and an increased number of plasma cells may indicate enhanced tumor immune response function, which can help stop the progression of tumors. Figure 7E, 7F displayed that 47 common immune checkpoint genes and 24 human leukocyte antigen (HLA) associated molecules were highly expressed in the high-risk group. B cells, mast cells, neutrophils, NK cells, type-II IFN response, and cytolytic activity were more stimulated in the low-risk group, whereas aDCs, IDCs, macrophage, Treg, and MHC class I were more stimulated in the high-risk group (Figure 7G). Tumor cells have developed various mechanisms to evade immune surveillance, including enhancing TMB, which can disguise the tumor cells from being recognized by the immune system. However, on the other hand, TMB has also been found to be a reliable predictor of the likelihood of an immunological response to the tumor, meaning that higher TMB levels may indicate a better likelihood of a successful immune attack against the tumor. The mutation landscape revealed that 159 (86.41%) of 184 samples in the high-risk group had mutations, with TP53 and CTNNB1 occupying the top two mutation frequencies, while 150 (84.75%) of 177 samples in the low-risk group did (Figure 7H, 7I). TMB was consistently considerably greater in the high-risk group compared to the low-risk group (Figure 7J).

**Figure 8:**
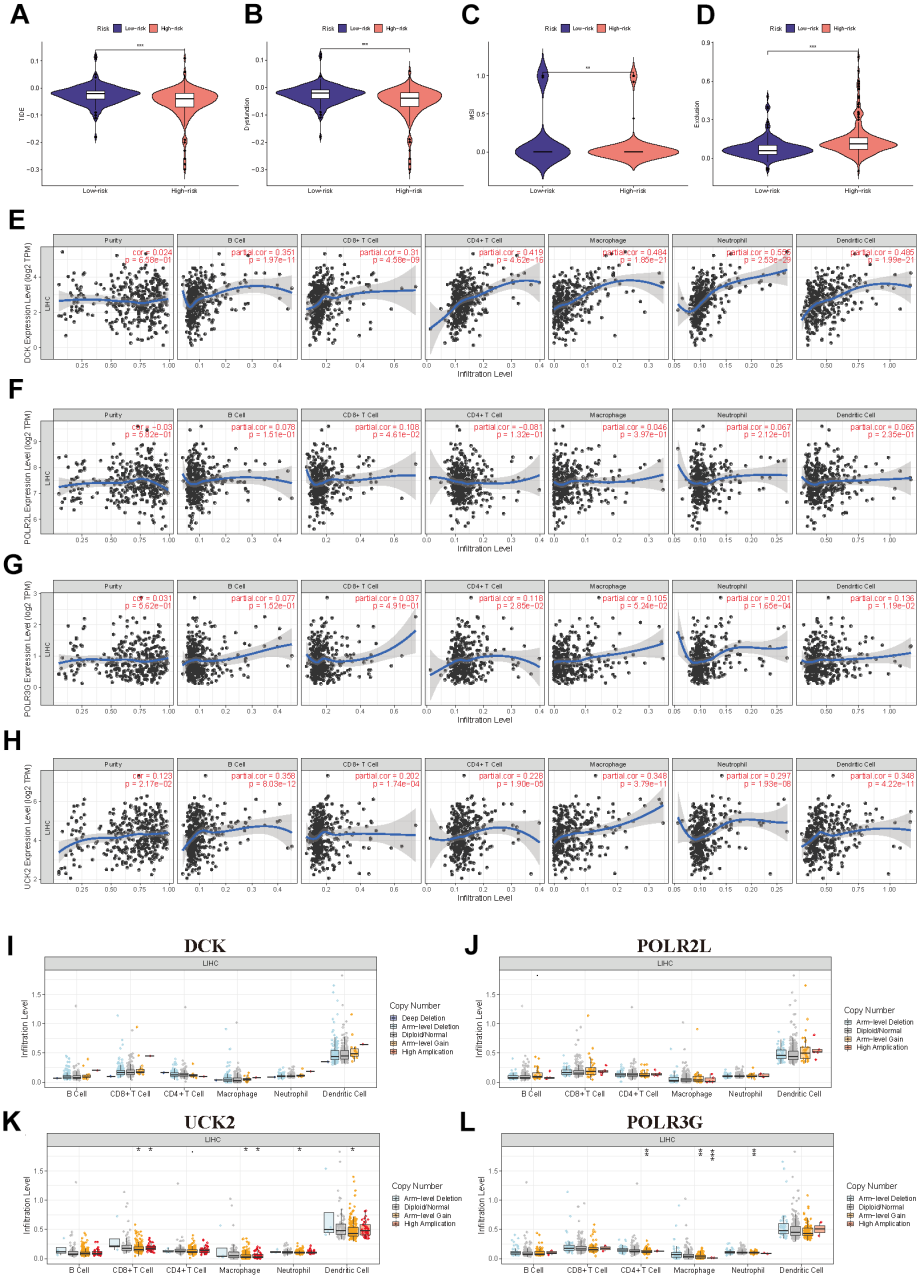
**Further exploration of the immune landscape in PMRS.** (**A**–**D**) TIDE analysis of the two risk groups. (**E**–**H**) The relation between the expression of four model genes and immune cell infiltration. (**I**–**L**) The relation between the mutation landscape of four model genes and immune cell infiltration. (*P<0.05, **P<0.01, ***P<0.001, ****P<0.0001).

Analysis of tumor immune dysfunction and exclusion (TIDE) includes the TIDE score, the Dysfunction score, the MSI score, and the Exclusion score. In our study, the TIDE score, Dysfunction score, and MSI score were higher in the low-risk group than in the high-risk group, whereas the Exclusion score was lower in the low-risk group than in the high-risk group, indicating the patient’s immune system in the low-risk group triggered a weaker reaction to the tumor, possibly due to the strong suppression of the immune cell (Figure 8A–8D). TIMER also investigated the relationship between immune cell infiltration and the expression levels of four model genes. Figure 8E–8H demonstrate that DCK and UCK2 were primarily associated with the infiltration of B cells, CD4+/CD8+ T cells, macrophages, neutrophils, and dendritic cells. In addition, we investigated the association between the level of infiltration of these immune cells and the mutational status of four model genes (Figure 8I–8L).

### Immunotherapy efficacy prediction

**Figure 9:**
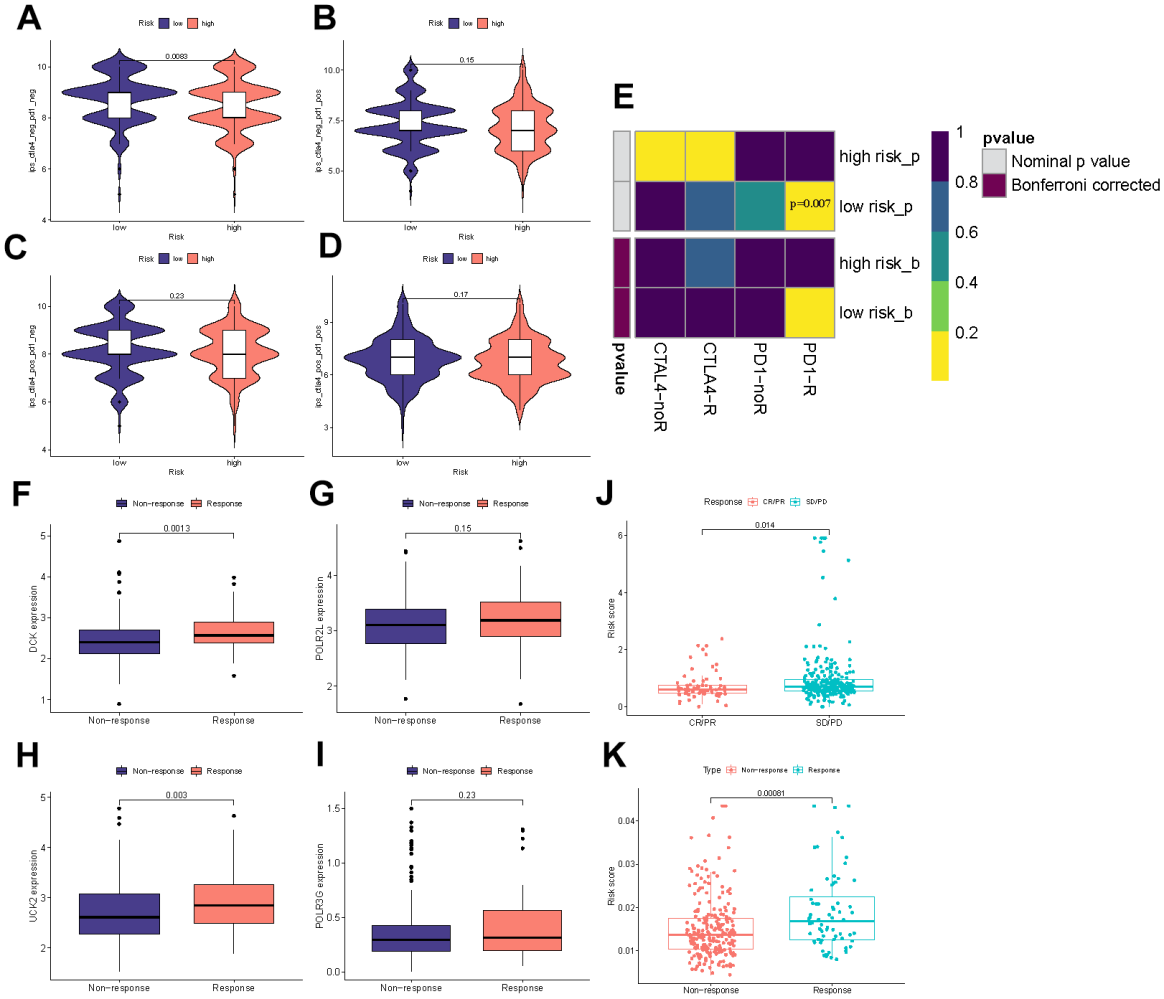
**Immunotherapy efficacy prediction.** (**A**–**D**) IPS comparisons between the two risk categories. (**E**) Submap analysis between the different groups. (**F**–**I**) The differential expression of four model genes between response and non-response groups. (**J**) Comparisons of the risk score in different responses to anti-PD-1/L1 immunotherapy in IMvigor210 cohort. (**K**) Differences in risk score between the response and non-response groups in the IMvigor210 cohort.

The immunotherapeutic response exhibited by the cohorts categorized as high-risk and low-risk was assessed using a combination of the IPS, Submap algorithms, and an external cohort undergoing analogous immunotherapy treatments. The fact that the percentage of ‘ips_ctla4_neg_pd1_neg’ is greater in the low-risk group than in the high-risk group suggests that patients in the low-risk group have a lesser expression of CTLA-4 and PD-1 immune checkpoint proteins in their tumor microenvironment. This often leads to reduced effectiveness of tumor immune surveillance. Therefore, patients in the low-risk group may respond better to immunotherapy and have a better prognosis compared to those in the high-risk group (Figure 9A–9D). Submap analysis yielded results uncovering the therapeutic response to anti-CTLA4 and anti-PD-1 immunotherapy among patients with HCC. The nominal P-value (P = 0.007) provided evidence supporting a greater likelihood of positive response to anti-PD-1 immunotherapy in the low-risk cohort relative to the high-risk group (Figure 9E). iMvigor210 cohort is designed to evaluate the efficacy and safety of atezolizumab, an immunotherapeutic drug used for the treatment of metastatic urothelial carcinoma (mUC). DCK and UCK2 in four model genes were highly expressed in the response group which referred to potentially serve as a predictive factor related to the response of tumor immune therapy (Figure 9F–9I). As the results in Figure 9J, 9K displayed, the Riskscore exhibits higher values in the response group and lower values in the no response group, as well as higher values in the SD/PD group and lower values in the CR/PR group. This observation commonly implies that the Riskscore can function as a stable and dependable indicator, thereby enhancing its capacity to effectively predict patient responsiveness to immunotherapy drugs.

### Estimation of drug sensitivity

**Figure 10:**
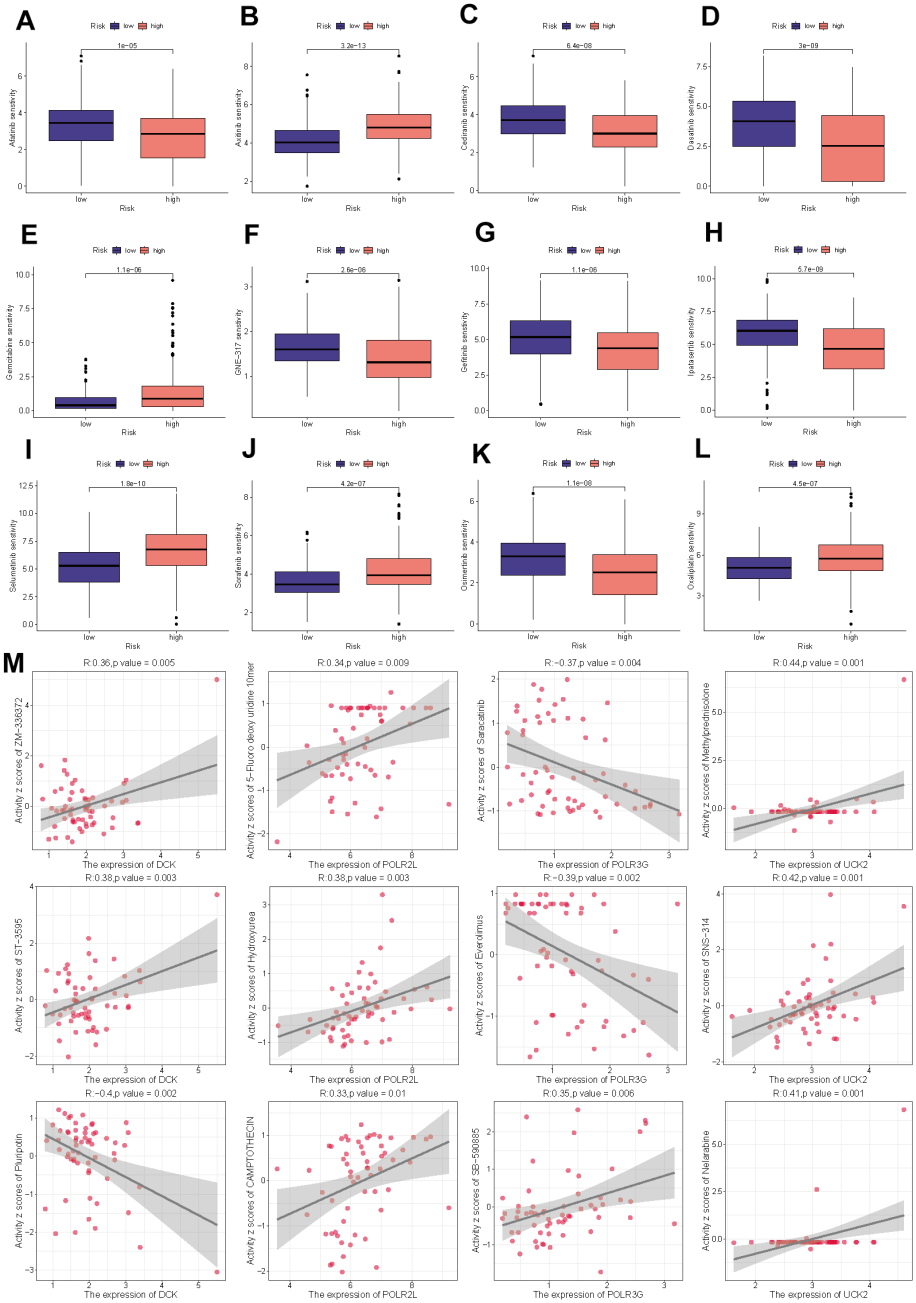
**Drug sensitivity analysis.** (**A**) Afatinib. (**B**) Axitinib. (**C**) Cediranib. (**D**) Dasatinib. (**E**) Gemcitabine. (**F**) GNE-317. (**G**) Gefitinib. (**H**) Ipatasertib. (**I**) Selumetinib. (**J**) Sorafenib. (**K**) Osimertinib. (**L**) Oxaliplatin. (**M**) The correlation between the drug sensitivity and expression levels of the four model genes.

To further examine the clinical applicability of PMRS for the precise treatment of HCC, we evaluated the therapeutic effectiveness of commonly prescribed chemotherapeutic agents in various risk categories. Based on the findings, low-risk individuals were more sensitive to Afatinib, Cediranib, Dasatinib, GNE-317, Gefitinib, Ipatasertib, and Osimertinib, whereas less responsive to Axitinib, Gemcitabine, Selumetinib, Sorafenib, and Oxaliplatin (Figure 10A–10L). Figure 10M demonstrates the selection and analysis of drugs that are sensitive to the expression of four model genes, which were screened through the CellMiner database.

### Analysis of PMRS in single cell level

**Figure 11:**
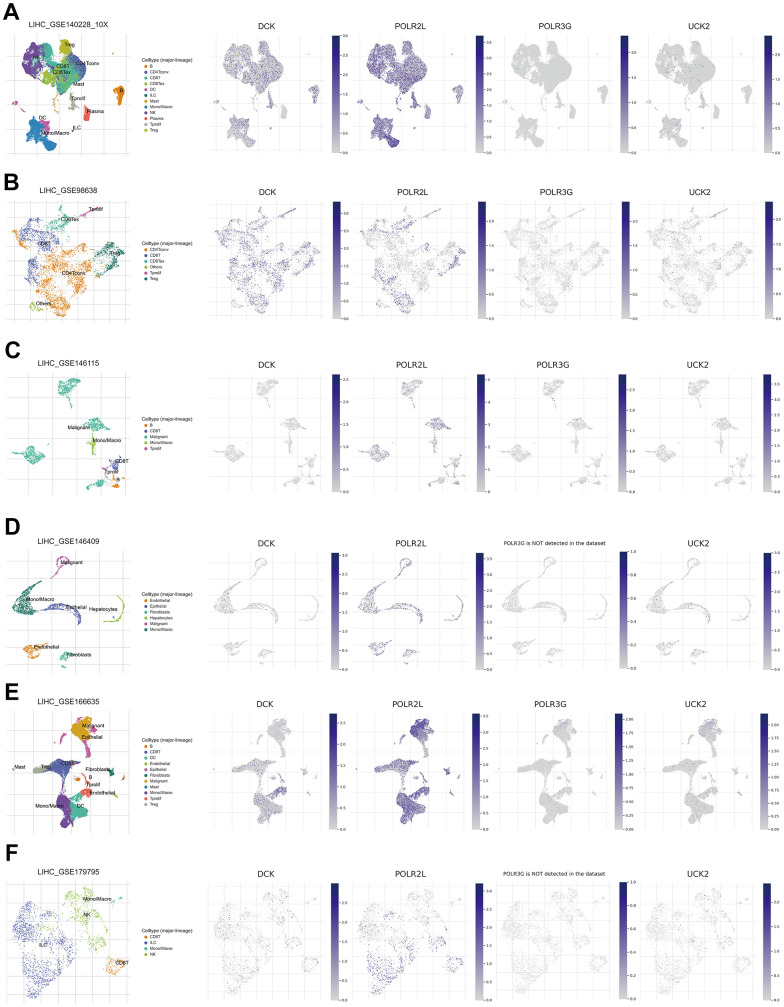
(**A**–**F**) Single-cell analysis of four model genes (DCK, POLR2L, POLR3G, UCK2) via TISCH based on GSE140228, GSE98638, GSE146115, GSE146409, GSE166635, GSE179795 datasets.

Tumor Immune Single Cell Hub (TISCH) is a massive managed database that incorporates nearly 2 million single-cell transcriptomic profiles. All the data were uniformly processed using a standardized methodology, which included quality control, eradication of batch effect, clustering, cell-type annotation, classification of malignant cells, variation in expression analysis, and functional enrichment analysis [28]. The scatter plots illustrated that DCK and POLR2L in the signature were significantly associated with tumor immune microenvironment through single cell analysis based on GSE140228, GSE98638, GSE146115, GSE146409, GSE166635, GSE179795 (Figure 11A–11F).

### The role of key gene DCK in hepatocellular carcinoma was verified *in vitro*

**Figure 12:**
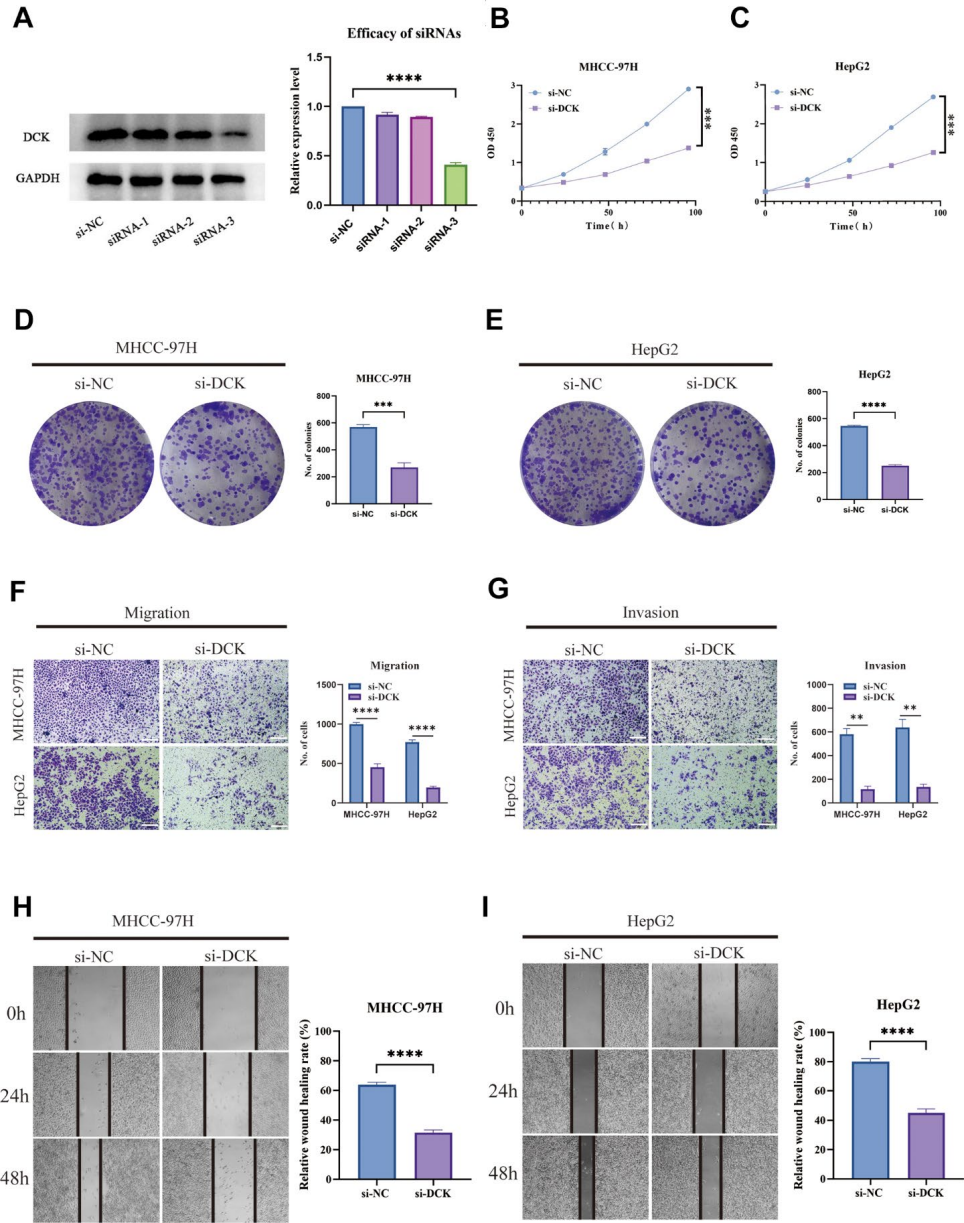
**Suppression of DCK inhibited the proliferative and migrative ability of LIHC cells.** (**A**) Transfected efficiency of three siRNAs targeting DCK. (**B**, **C**) The activity of MHCC-97H and HepG2 cell lines was dramatically inhibited following DCK knockdown. (**D**, **E**) Colony formation assay revealed that the capacity of these two cell lines to generate colonies was significantly decreased after DCK knockdown. (**F**–**I**) The migrative and invasive ability of MHCC-97H and HepG2 cell lines was considerably suppressed in the transwell assay and wound healing experiment. (*P<0.05, **P<0.01, ***P<0.001, ****P<0.0001).

In light of the previous findings, we conducted cell experiments to validate the use of PMRS. Firstly, the siRNAs targeting the expression of DCK were evaluated using Western blot assay. The results indicated that siRNA-3 exhibited the highest efficiency in downregulating DCK protein levels, and therefore was selected for further analysis conducted (Figure 12A). The activity of hepatocellular carcinoma cells was dramatically inhibited following DCK knockdown in MHCC-97H and HepG2 cell lines (Figure 12B, 12C). Afterward, the colony formation assays showed that the capacity of these two cell lines to generate colonies was significantly decreased after DCK knockdown (Figure 12D, 12E). The results of wound healing and transwell assays demonstrated a significant reduction in the migration and invasion capacity of hepatocellular carcinoma cells following DCK knockdown through transwell assay (Figure 12F, 12G) and wound healing assay (Figure 12H, 12I).

## DISCUSSION

Pyrimidines are a class of organic compounds with a heterocyclic ring structure containing two nitrogen atoms at opposite positions in the ring. They serve as key building blocks for DNA and RNA, along with purines. Additionally, pyrimidines are involved in cellular metabolism, serving as precursors for the biosynthesis of important biomolecules such as nucleotides, coenzymes, and heme. The core enzyme CAD, which is involved in the de novo synthesis of pyrimidines, is essential for maintaining proper levels of pyrimidine nucleotides in cells [30] and for anti-tumor immunomodulatory effects [31, 32]. Pyrimidine metabolism is a complicated enzymatic network that involves nucleoside salvage, de novo nucleotide synthesis, and the breakdown of pyrimidines. In contrast to resting cells, cancer cells depend on the de novo pathway to generate a constant supply of deoxyribonucleoside triphosphates (dNTPs) in order to sustain uncontrolled tumor growth [33].

Hepatocellular carcinoma (HCC) is a major contributor to cancer-related morbidity and mortality worldwide. While traditional clinicopathological factors such as tumor size, stage, and degree of differentiation are primary indicators for predicting prognosis and guiding treatment decisions, they may not accurately reflect prognosis due to the high heterogeneity of the malignancy. Therefore, the identification and validation of specific and robust liver cancer biomarkers hold great significance for the early detection, diagnosis, prognosis, and prevention of tumor progression by utilizing multi-omics technology to comprehensively research the immune microenvironment, gut microbiota, liquid biopsy, and individualized treatments [34]. Furthermore, certain molecular risk models that have not been extensively implemented in clinical management have the potential to accurately predict HCC, including ferroptosis phenotype-related, cuproptosis-related, and hypoxia-related prognostic signatures [35–37]. The role of pyrimidine metabolism in HCC, however, is not well understood. In this study, we developed a signature based on pyrimidine metabolism that may be used for risk assessment, prognosis, and treatment planning in patients with HCC, thereby facilitating effective clinical decision-making.

As models based on single genes lack robustness and stability, we utilized machine learning methods to identify a 4-gene (DCK, UCK2, POLR2L, and POLR3G) signature related to pyrimidine metabolism. By incorporating clinical indicators with the signature, we developed a comprehensive nomogram that enables accurate predictions. Notably, the relevance of the risk score was shown by the fact that it was responsible for a significant amount of the overall score generated by the model.

The tumor microenvironment (TME) comprises diverse types of stromal, innate, and adaptive immune cells. These populations of cells often engage in cooperative or competitive interactions that facilitate tumor growth, progression, metastasis, as well as evasion from the host immune system [38, 39]. The stromal score is derived from the expression levels of genes associated with non-cancerous stromal cells surrounding tumors [40]. A higher stromal score typically indicates a greater amount of stromal cell infiltration within the tumor microenvironment, which may suggest a better prognosis due to an increased presence of immune cells that can target cancer cells [41]. However, it is important to note that the interpretation of stromal scores must be considered in conjunction with other clinical factors and should not be used as the sole determinant for treatment decisions. In addition, the low-risk group infiltrated more plasma cells and fewer M0 macrophages. Across diverse cancer types, the presence of Mϕ infiltration within the tumor microenvironment is frequently associated with advanced clinical stage and unfavorable prognosis [42, 43]. Hence, strategies to specifically target these Mϕ subpopulations may prove instrumental in advancing the field of tumor immunotherapy, and ultimately improving patient outcomes. M0 macrophages are a type of immune cell that can be found in the tumor microenvironment and their abundance or ratio with other macrophage subtypes can potentially impact tumor progression. An increased presence of M0 macrophages may suggest a pro-inflammatory environment within the tumor microenvironment, which has been associated with an increased risk of tumor progression [44, 45].

Researchers have discovered that cancer cells may escape immune monitoring via a variety of strategies, one of which is activating a pathway known as the immunological checkpoint. Immune checkpoint inhibitors (ICIs) function by reversing immunological tolerance to tumors, overcoming tumor cell-mediated immune suppression, restoring anti-cancer immunity, and facilitating the clearance of tumor cells [46, 47]. In the majority of HCC patients, the PD-1/PD-L1 pathway alone may not suffice as the rate-limiting factor in counteracting tumor-mediated immune suppression. Hence, therapeutic intervention based solely on blocking the PD-1/PD-L1 axis may not be adequate for eliciting an effective anti-tumor immune response [48]. In this context, combination therapy that can act upon multiple pathways may present itself as a more viable strategy for enhancing the overall treatment efficacy. A meta-analysis revealed that despite an overall objective response rate that remained below 30%, the administration of PD-1/PD-L1 inhibitors in combination with anti-angiogenic agents and dual immunotherapy resulted in significantly increased survival time when compared to Sorafenib [49]. Our investigation evaluated the immunotherapy response across distinct risk groups based on the pyrimidine metabolism-related signature. Patients in the low-risk category were shown to have a higher likelihood of benefiting from immunotherapy treatment. This finding, which highlights the strong predictive value of the detected signature, was discovered via the use of various algorithms and an independent cohort. In addition, a complete analysis of this gene signature was able to efficiently differentiate between several subtypes of HCC cells, which demonstrates its potential for use in future therapeutic approaches.

It should be noted that our study carried several limitations. Firstly, the utilization of an existing public database as the foundation for our research warrants the need for subsequent validation through multi-centric prospective trials. Secondly, the existence of unknown interactions between gene products and genes present in the identified signature can have significant implications for both physiology and pathology. Therefore, further probing through complementary experiments conducted using *in vivo* models is necessary to get better insights into the underlying mechanisms of the gene signature in question.
